# Coherent Thermal Conduction in Silicon Nanowires with Periodic Wings

**DOI:** 10.3390/nano9020142

**Published:** 2019-01-22

**Authors:** Roman Anufriev, Masahiro Nomura

**Affiliations:** 1 Institute of Industrial Science, University of Tokyo, Tokyo 153-8505, Japan; nomura@iis.u-tokyo.ac.jp; 2 PRESTO, Japan Science and Technology Agency, Saitama 332-0012, Japan

**Keywords:** phononic crystals, nanowires, thermal conductivity, silicon

## Abstract

Artificial periodic nanostructures, known as phononic crystals, promise to control the thermal properties of nanostructures in the coherent regime, which can be achieved in semiconductors at low temperatures. Here, we study coherent thermal conduction in silicon nanowires with added periodic wings at sub-Kelvin temperature. Our simulations show that the added periodic wings flatten the phonon dispersion and thus reduce the thermal conductance. We investigate the dependence of this reduction on the size of the wings and conclude that the reduction is mainly caused by the periodicity of the wings, rather than by local resonances in them. These findings help to better understand the mechanisms controlling coherent heat conduction in periodic resonant nanostructures.

## 1. Introduction

Phononic crystals are a class of artificial metamaterials that aim to control phonon transport in the coherent regime [1,2]. In this regime, phonons behave like elastic waves and experience interference due to reflections from the structure boundaries. The interference changes the phonon dispersion relation which determines thermal properties of the structure. Thus, phononic crystals can control thermal properties of semiconductor nanostructures and find applications in heat conduction engineering [3]⁠. 

Experiments have demonstrated that such heat conduction engineering is indeed possible at low temperatures in membranes with two-dimensional arrays of holes [4,5]⁠. In these membranes, the thermal properties can be tuned using periodicity of the holes [4,6,7,8]⁠. Lately, theoreticians proposed to replace holes with pillar-like resonators, which would allow finer tuning of the thermal properties via resonator size [9]⁠. However, the fabrication of membranes with two-dimensional arrays of resonators is challenging, because the process needs to produce low surface roughness and low contact resistance at the resonator–membrane interface [10,11]⁠. 

Alternatively, instead of the vertical pillar-like resonators, the resonators can be fabricated in the plane of the wafer. Following this route, experimental works studied one-dimensional phononic structures consisting of nanowires with periodic constrictions [12,13,14]⁠ or side wings [13,15]⁠⁠. Experiments demonstrated that such periodic nanowires have lower thermal conductivity than the pristine nanowires [12,13,14,16]. Some authors [16]⁠⁠ attributed this reduction in thermal conductivity to the coherent effects even at room temperature, while others [14]⁠ attributed it to the incoherent surface scattering of phonons at low temperatures. Such diverse interpretations of the experimental results reflect incomplete theoretical understanding of the coherent heat conduction in one-dimensional phononic nanostructures.

Here, we theoretically study coherent heat conduction in nanowires with additional side wings. We demonstrate how the wings and their dimensions impact the thermal conductance of nanowires in the coherent regime and discuss the mechanisms of the observed reduction in the thermal conductance.

## 2. Materials and Methods

**Figure 1:**
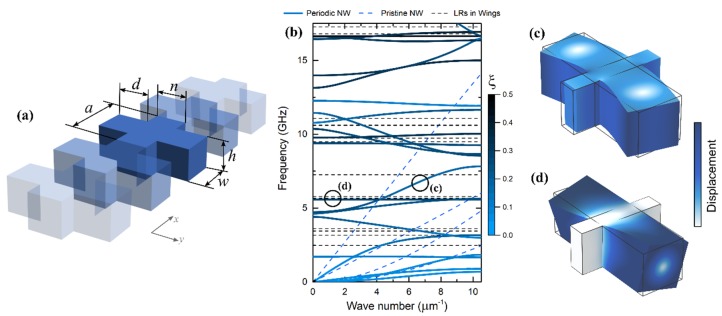
(**a**) Schematic of a simulated silicon nanowire with wings (*a* = 300 nm, *n* = 60 nm, *w* = 150 nm, *d* = 200 nm, and *h* = 145 nm). (**b**) Phonon dispersion of the nanowire with wings plotted with the dispersion of pristine nanowire (blue dashed lines) and eigenfrequencies (LR) of the wings (black dashed line). The color of the dispersion branches indicates the physical location of the mode: Blue shows the modes inside the nanowire and black shows the modes inside the wings. (**c**,**d**) Displacement fields of the modes indicated in (**b**) by circles.

To simulate heat conduction in the coherent regime, we assumed the fully elastic behavior of phonons. Under this assumption, we could obtain phonon dispersion by numerically solving the elastodynamic wave equation for the structure of a given geometry [4,6,8]. For this purpose, we used the finite element method implemented in Comsol Multiphysics. Figure 1a illustrates the simulated structure. The periodic Floquet boundary conditions were applied along the *y* axis. The material and dimensions were chosen to match the samples experimentally studied by Maire et al. [13]⁠ (i.e., silicon with *a* = 300 nm, *n* = 60 nm, and *h* = 145 nm).

The eigenfrequencies (ω) of the structure were calculated for one hundred wave vectors (*k*) in the direction parallel to the nanowire axis. To sort the obtained eigenfrequencies into continuous dispersion branches, we assumed that the branches continued across the apparent band intersections, which was the case for the vast majority of the intersections in the Γ–X direction [17]⁠. To study the thermal properties of the structure, we calculated the thermal conductance (*G*) as:

(1) $$G\propto \underset{m}{\sum }\int_{\Gamma }^{X}\hbar \omega_{m}\frac{d\omega_{m}}{dk}\frac{df}{dT}dk$$

where *m* is the number of the phonon mode and *f* is the Bose–Einstein distribution. In a reasonable time, for each wave vector (*k*), the solver can find only a several hundred eigenfrequencies (ω), which cover the range < 100 GHz. Thus, we could calculate the thermal conductance only at *T* = 0.5 K. In this calculation, we assumed the uncertainty of 5% for the thermal conductance (*G*) values and, consequently, 7% for the relative *G*/*G*_NW_ values. Details of this simulation technique can be found in our previous work [9]⁠.

## 3. Results and Discussion

Figure 1b shows the phonon dispersion of the nanowire with the wings (solid lines), together with the four branches of original phonon dispersion in a pristine nanowire without wings (dashed blue lines) and eigenfrequencies (local resonances) of the wings (dashed black lines). The dispersion was generally flattened, as compared to the branches of the pristine nanowire. This flattening was caused by two phenomena: First, the interference due to periodicity of the wings [18]⁠; second, the local resonances in the wings [19,20]⁠. The impact of periodicity can be clearly seen in the flattening of the first three branches but is less obvious for higher frequency branches. The impact of local resonances can be seen as flat bands around 6, 9, and 17 GHz, close to resonant frequencies of the wings. To distinguish these two phenomena more clearly, the color of the dispersion branches shows the center of elastic energy (*ξ*) in the *y* axis, calculated as:

(2) $$\xi =\frac{1}{n/2+d}\frac{\int_{V}F\left|y\right|dV}{\int_{V}FdV}$$

where *F* is elastic energy density and the integral is evaluated over the volume (*V*) of a unit cell [21,22]⁠⁠. Thus, the lighter branches contained regular non-localized modes (Figure 1c), whereas dark flatter branches contained the modes localized in the wings (Figure 1d).

**Figure 2:**
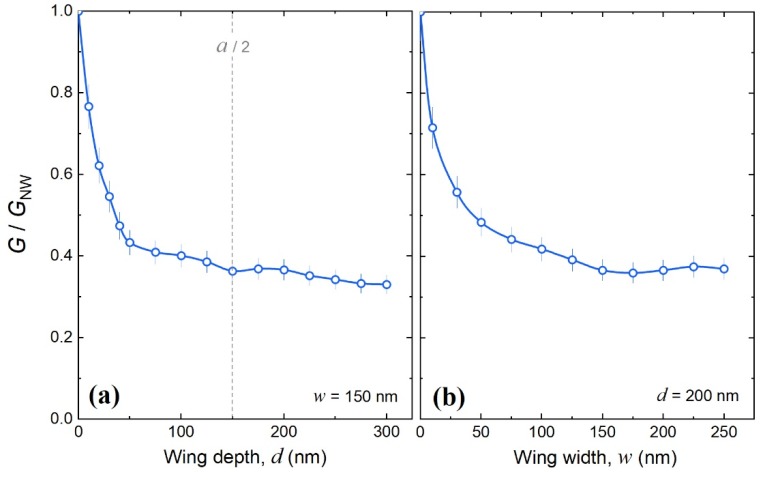
The relative thermal conductance of nanowires with wings (*a* = 300 nm, *n* = 60 nm, and *h* = 145 nm) as a function of (**a**) depth and (**b**) width of the wings; simulated at 0.5 K.

To study the impact of these modifications of phonon dispersion on heat conduction, we plotted the relative thermal conductance *G*/*G*_NW_, obtained as the thermal conductance of the nanowire with wings (*G*) divided by that of a nanowire without wings (*G*_NW_). Figure 2a shows the relative thermal conductance (*G*/*G*_NW_) as a function of the wing depth (*d*). The thermal conductance quickly dropped by 60% as soon as short 50-nm-deep wings were added to the nanowire. But, as the wings become deeper, the thermal conductance decreased at a much slower rate. Similar dependence on the resonator length was observed for two-dimensional phononic membranes with pillars [22]⁠ and graphene ribbons with wings [23]⁠. Interestingly, at the wing depth equal to the wing widths (*w*) and a half period (*a*/2), the dependence showed a small dip, caused by the opening of additional phononic bandgaps due to the additional symmetry. 

Figure 2b shows the dependence on the wing width (*w*). The thermal conductance first dropped by 50%, even for relatively narrow wings (*w* < 50 nm), and then saturated as the wings became wider. Thus, the width and depth dependencies were similar, and both showed that the reduction in thermal conductance was caused by the mere addition of the wings and did not strongly depend on the wing size.

These observations hold the key to the origin of the thermal conductance reduction in one-dimensional phononic nanostructures. Indeed, the addition of the wings introduced periodicity that caused periodic phonon interference, whereas the wing size controlled the spectral density of local resonances. Thus, the thermal conductance seemed to be mainly impacted by the periodicity rather than by local resonances. At least, an increase in the number of local resonances did not lead to a stronger reduction in the thermal conductance.

## 4. Conclusions

We investigated heat conduction in silicon nanowires with periodic wings in a purely coherent regime at 0.5 K. Our simulations show that the wings change the phonon dispersion of nanowires and thus reduce the thermal conductance. The dependencies of the thermal conductance on the width and depth of the wings suggest that the main impact probably comes from the periodicity of the wings. The impact of local resonances seems weaker, or at least does not depend on their number. These conclusions imply that even short wings are sufficient for applications requiring the suppression of thermal conductance. Fundamentally, these findings deepen the understanding of coherent control of heat conduction and clarify the impact of different types of phonon interference in periodic resonant nanostructures.
